# Reconstruction of segmental bone defect of long bones after tumor resection by devitalized tumor-bearing bone

**DOI:** 10.1186/s12957-015-0694-3

**Published:** 2015-09-24

**Authors:** Huayi Qu, Wei Guo, Rongli Yang, Dasen Li, Shun Tang, Yi Yang, Sen Dong, Jie Zang

**Affiliations:** Musculoskeletal Tumor Center, People’s Hospital, Peking University, Xizhimen Nan 11#, Xicheng District, Beijing, 100044 China

**Keywords:** Intercalary bone defect, Bone tumor, Devitalization, Reconstruction

## Abstract

**Background:**

The reconstruction of an intercalary bone defect after a tumor resection of a long bone remains a challenge to orthopedic surgeons. Though several methods have been adopted to enhance the union of long segmental allografts or retrieved segmental autografts to the host bones, still more progresses are required to achieve a better union rate. Several methods have been adopted to devitalize tumor bone for recycling usage, and the results varied. We describe our experiences of using devitalized tumor-bearing bones for the repairing of segmental defects after tumor resection.

**Methods:**

Twenty-seven eligible patients treated from February 2004 to May 2012 were included. The segmental tumor bone (mean length, 14 cm) was resected, and then devitalized in 20 % sterile saline at 65 °C for 30 min after the tumor tissue was removed. The devitalized bone was implanted back into the defect by using nails or plates.

**Results:**

Complete healing of 50 osteotomy ends was achieved at a median time of 11 months (interquartile range (IQR) 9–13 months). Major complications included bone nonunion in four bone junctions (7.4 %), devitalized bone fracture in one patient (3.7 %), deep infection in three patients (11.1 %), and fixation failure in two patients (7.4 %). The bone union rates at 1 and 2 years were 74.1 and 92.6 %, respectively. The average functional score according to the Musculoskeletal Tumor Society (MSTS) 93 scoring system was 93 % (IQR 80–96.7 %).

**Conclusions:**

Incubation in 20 % sterile saline at 65 °C for 30 min is an effective method of devitalization of tumor-bearing bone. The retrieved bone graft may provide as a less expensive alternative for limb salvage. The structural bone and the preserved osteoinductivity of protein may improve bone union.

## Background

Most of the limbs can be salvaged for those patients with a bone tumor of the extremity, due to multidisciplinary advances in orthopedic oncology [1]. Advances in imaging techniques allow for a more precise preoperative determination of the tumor extent [2], which allows a more accurate resection of the tumor, especially with the help of advanced techniques such as an image-guided navigation system [3]. A more accurate resection of the tumor allows a nearby joint to be preserved in some patients with a tumor adjacent to it without increasing the local recurrence rate. This means that patients may obtain a better functional result than with a routine endoprosthesis replacement.

Several surgical techniques may be applied to reconstruct large defects of long bones after tumor resection. These techniques include intercalary prostheses [4], allografts [5], devitalized tumor-bearing bones [6], and the induced membrane technique [7]. Each technique has its advantages and disadvantages, making selection of the most appropriate technique complex. The shape of the devitalized bone and the osteoinductivity of proteins in the bone may be preserved by several devitalization methods, which may facilitate bone union and make these methods favorable options for intercalary bone reconstruction [6].

Our previous study demonstrated that tumor cells in the tumor-bearing bone may be devitalized effectively by incubation in 20 % sterile hypertonic saline at 65 °C for 30 min [8]. Incubation of the tumor-bearing bone at 65 °C for 30 min, or pasteurization, is an effective method of devitalization [6]. We choose 20 % saline over physiological saline as the devitalizing reagent because a high concentration of salt may increase the thermal stability of proteins, which may protect the protein activity during devitalizing [9, 10].

We retrospectively reviewed 27 cases of intercalary bone defects that had been reconstructed with devitalized tumor-bearing bones from February 2004 to May 2012. The aims of the study were to analyze the clinical results of the intercalary tumor-bearing bone devitalized by our method and used to repair the massive segmental defects following tumor resection of long bones and to observe the time required for bone integration and the incidence of any ensuing complications.

## Methods

**Fig. 1:**
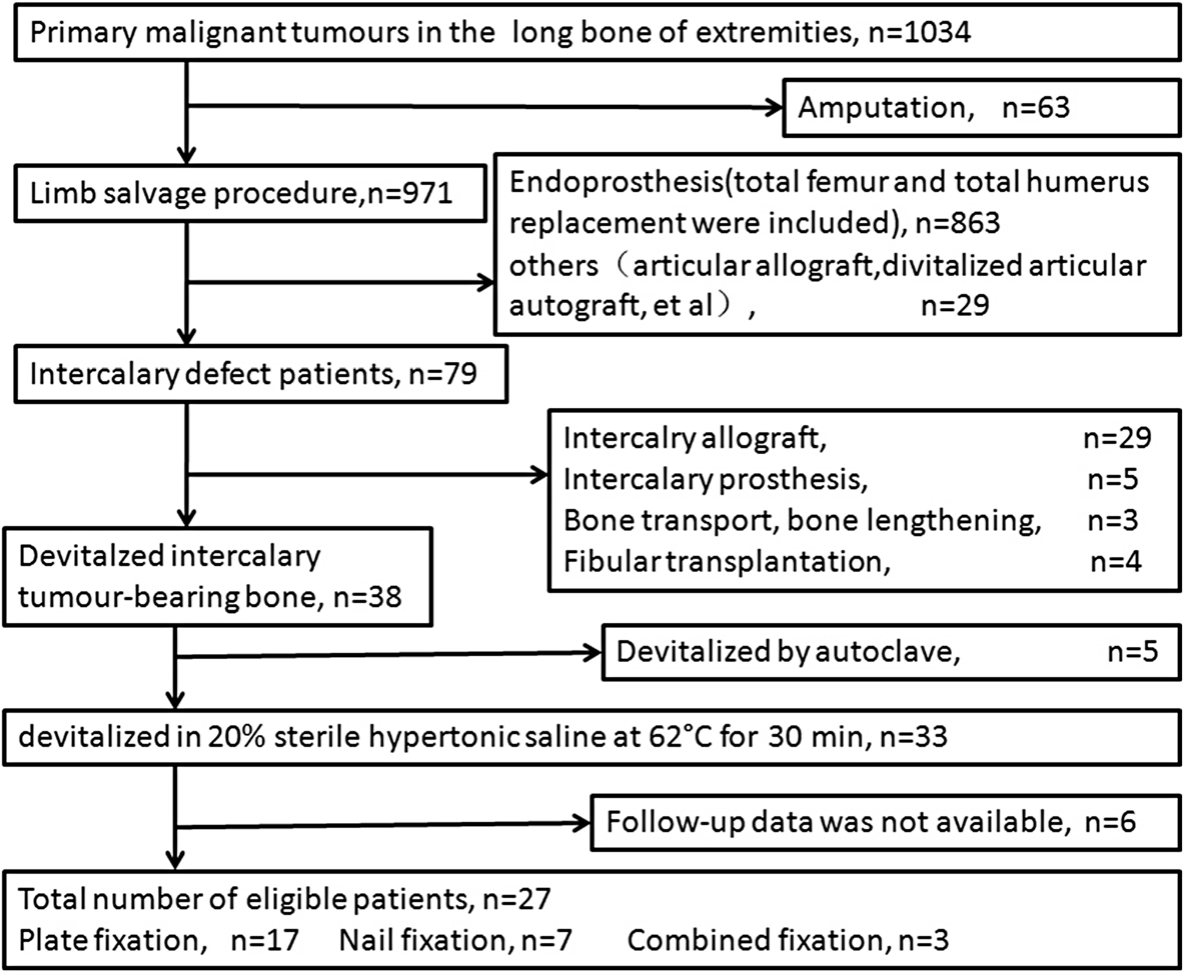
A flow-chart showing the selection of patients in this study when applying our inclusion criteria

**Fig. 2:**
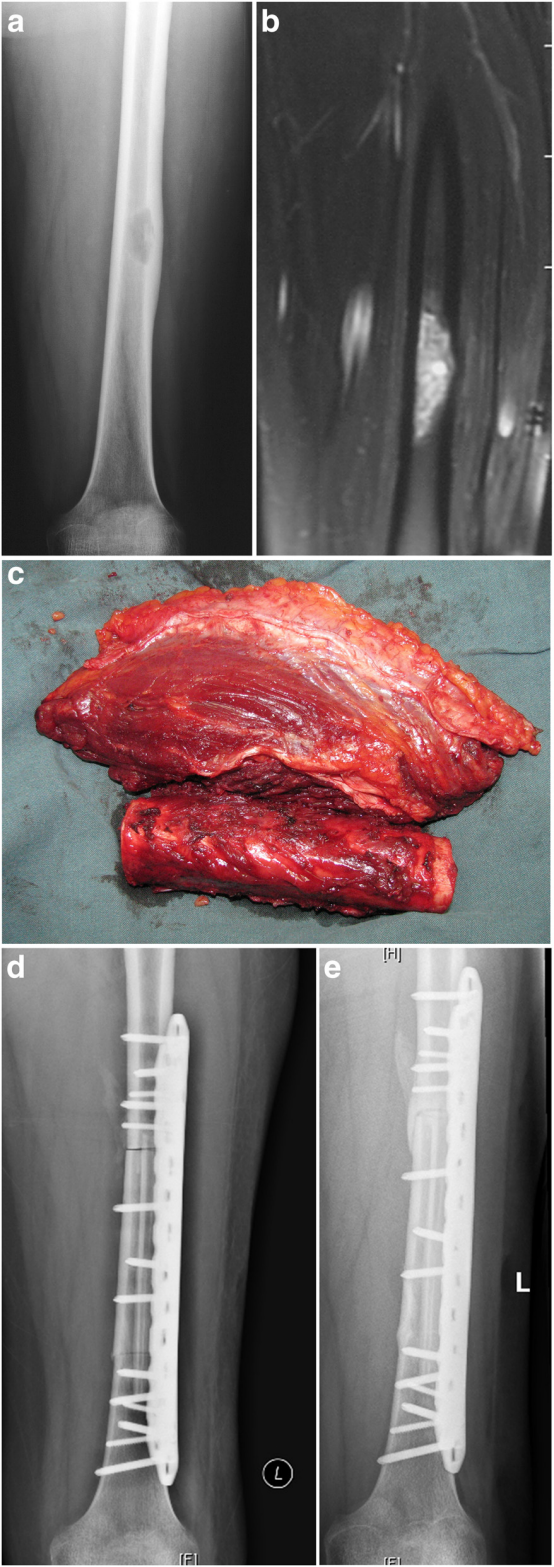
A 26-year-old male patient with an Ewing sarcoma in the middle part of the right femur. **a** Radiograph showed a lytic lesion in the diaphysial region of the right femur. **b** MRI showed the lesion was about 8 cm. **c** A 12-cm segmental bone was resected, the soft tissue was removed, and the structural bone and the periosteum were preserved for divitalization. **d** Radiograph 2 weeks after the reconstruction showed that the devitalized bone fit the defect perfectly. **e** Continuous callus at the proximal junction and a perfect union at the distal junction were observed 8 months after the reconstruction

**Fig. 3:**
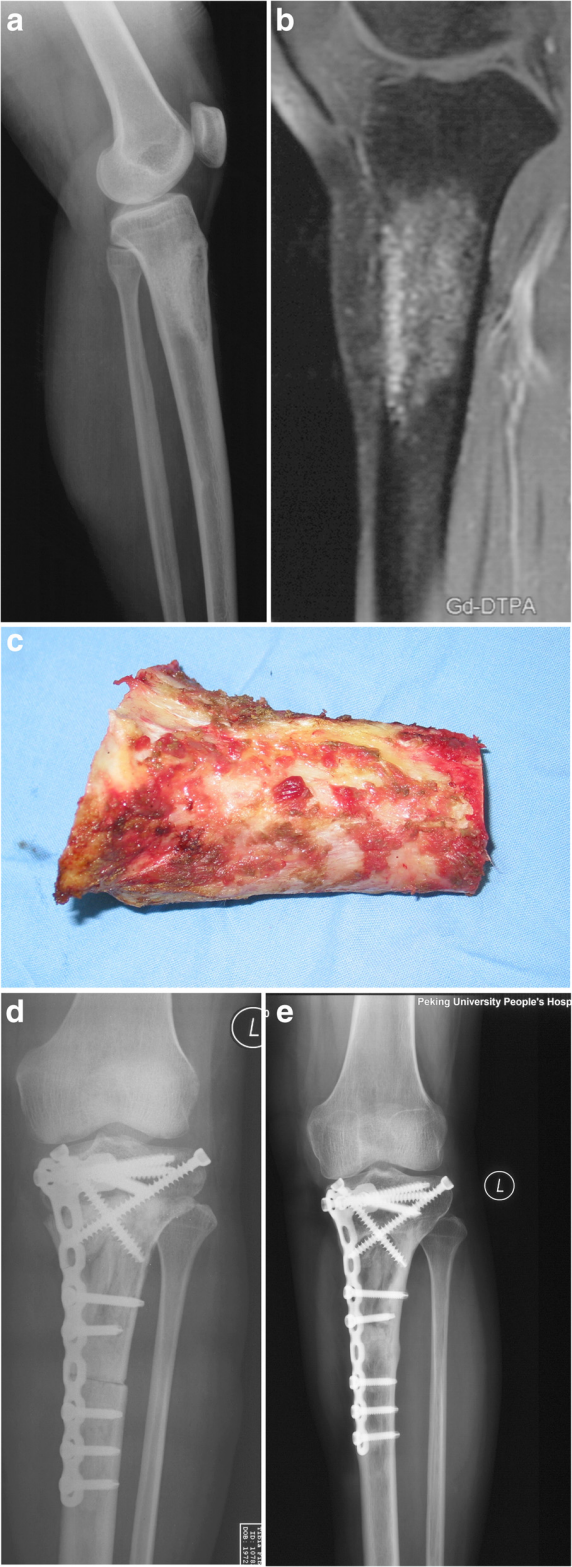
A 42-year-old patient with an adamantinoma of the left proximal tibia. **a** X-ray showed the lesion located at the proximal part of the left tibia. **b** MRI showed the knee joint was not involved by the tumor. **c** The structural bone and the periosteum were preserved after the tumor was cleared away. **d** X-ray showed the devitalized bone matched well with the host bones after the operation. **e** X-ray showed the cutting lines was disappeared, and the devitalized bone integrated well with the host bone at each ends 11 months after the operation

Twenty-seven patients who underwent tumor resection, bone devitalization, and reconstruction of long bones between February 2004 and May 2012 were included in this study. The inclusion criteria were as follows: (1) a primary long-bone tumor that required massive segmental resection to cure the disease, in which the joint on each end was preserved to maintain function (Figs. 2a and 3a); (2) no prior treatment; (3) availability of complete imaging data, pathology results, and follow-up information; and (4) a minimum of 12 months of follow-up after surgery. A flow chart of how the patients were recruited is summarized in Fig. 1. Informed consent was obtained from all patients.

The average duration of the follow-up was 62.8 months (range 15–116 months). Sixteen males and 11 females were included in this study, with a mean age of 28.3 years (range 9–73 years). The histological diagnoses were osteosarcoma (14 patients), Ewing’s sarcoma (six patients), chondrosarcoma (three patients), undifferentiated high-grade pleomorphic sarcoma (three patients), and adamantinoma (one patient). The tumors were located in the distal femur (14 patients), proximal tibia (eight patients), proximal humerus (three patients), distal radius (one patient), and proximal ulna (one patient).

The preoperative work-up comprised a patient history and clinical examination, routine laboratory tests, plain radiography, bone scanning, and computed tomography of the affected limb. Magnetic resonance imaging (MRI) of the affected limb was done to determine the resection site on the bone (Figs. 2b and 3b). Eighteen patients (12 with osteosarcoma and six with Ewing’s sarcoma) received neoadjuvant chemotherapy. An additional work-up of the patients as indicated above was performed to obtain a postoperative evaluation. According to the Enneking’s staging system of bone and soft tissue tumors, the patients were classified as stage IIB (23 patients), stage IIA (three patients), or stage IA (one patient).

An adequate resection margin was obtained by following standard oncology principles. The average resection length of the affected bone was 14 cm (range 7–25 cm). Intramedullary nails or plates were applied to fix the devitalized bone. In six patients, a free fibula graft was inserted in the devitalized bone with each end 1 cm longer than the devitalized bone as an additional strategy to enhance union because the fibula may offer extra bone stock and live cells to facilitate bone union. Four patients received allograft chip filling inserted into the intramedullary cavity to increase the bone stock. Two patients with undifferentiated high-grade pleomorphic sarcoma underwent postoperative chemotherapy, and one patient received radiation after surgery.

The bone tumor was resected by following standard oncology principles. During the preparation of the devitalization of the bone, the surrounding soft tissues and any tumor mass protruding out of the bone were removed and the periosteum around the bone was preserved carefully, especially at both ends of the intercalary bones. The tumor in the medullary cavity and the eroded cortex bone were removed (Figs. 2c and 3c). The bony segment was then incubated in 20 % sterile saline at 65 °C for 30 min. The retrieved bone was ready to be implanted after washing in normal saline at 37 °C.

The patients received continuous passive motion for 2 days after the operation to restore limb function and were allowed to perform partial weight-bearing exercise with a brace after 2 weeks. A follow-up was conducted every 3 months for 3 years, every 6 months for another 2 years, and then once each year. Bone union was assumed when the patient had no pain on weight-bearing and either the osteotomy line was no longer visible or there were bridging calluses on two orthogonal radiographic views. The limb function was evaluated in accordance with the Musculoskeletal Tumor Society (MSTS) system [11].

Statistical analyses were performed using SPSS software version 16.0. Kaplan–Meier analysis was used to analyze the survival status of the patients.

## Results

The main complications included bone nonunion, devitalized bone fracture, hardware failure, and deep infection. Bone union was achieved in 50 bone junctions out of 54 junctions after a median time of 11 months (interquartile range (IQR) 9–13 months).

Nonunion occurred in four bone junctions (7.4 %). One patient with nonunion in the proximal humerus received iliac bone grafting, and bone union was found after 9 months. An above-knee amputation was performed in another patient because the tibial nerve and popliteal vessels were trapped by a recurring tumor. One patient required removal of the devitalized bone and replacement with an endoprosthesis. In one patient who developed a pulmonary metastasis, the nonunion in the femur was left untreated. Devitalized bone and intramedullary nail fracture occurred in one patient (3.7 %) 18 months after the operation. An intramedullary nail and iliac bone grafting were used to treat the fracture, but this ended up causing bone absorption around the fracture. A proximal femoral endoprosthesis replacement was finally performed to reconstruct the limb function. Two patients with plate fixation developed hardware failure—one in the second and the other in the fourth month after the primary surgery. Reoperations were performed to restore the bone integrity by securing the devitalized bone with longer plates and cancellous bone grafting. Autograft or allograft chips were used in ten patients, but there is no significant difference between the patients with grafting and those without about union rate and union time.

Deep infection was found in three patients (11.1 %). Thorough debridement, gastrocnemius muscle flap transfer, and lavage of the wound were performed in one patient, and the bone graft and fixation were left in situ. Antibiotics were administered for 1 month according to an antibiotic susceptibility test, and bone union was achieved 1 year later. One patient with a femoral devitalized bone reconstruction experienced a deep infection with extensive bone absorption of the devitalized bone 3 months after the operation. The infection was controlled by the removal of the plate and devitalization of the bone, debridement of the wound, and placement of an antibiotic polymethyl methacrylate spacer. A distal femoral endoprosthesis replacement was then performed. One patient developed a deep infection 8 months after the operation. Solid union was detected during debridement and lavage, and the plate was removed and an external bracer was used to protect the limb for 3 months. No signs of infection were detected during the 65-month follow-up. Devitalized bone was removed because of tumor recurrence, infection, or nonunion after fracture in five patients, and 81.5 % of the devitalized bones were still in place at the last follow-up.

Recurrence developed in four patients (14.8 %). The recurrence was in the surrounding soft tissues in three patients, and in the residual bone in one patient. Above-knee amputation was performed in two patients because the neurovascular bundle was trapped by a recurrent tumor. One patient with a recurrent tumor in the soft tissues received local resection of the tumor. Endoprosthesis replacement was performed in the patient with residual bone recurrence. Four patients died of their disease during the follow-up, one patient lives with the disease, and one patient died of an unrelated disease. The 3- and 5-year postoperative survival rates according to the Kaplan–Meier survival analysis were 92.1 ± 5.4 and 86.4 ± 7.5 %, respectively. The average functional score according to the MSTS 93 scoring system was 93 % (IQR 80–96.7 %).

## Discussion

Although the metaphysis is the most common location of bone tumors, a number of tumors also originate from the diaphysial and metadiaphysial regions of long bones (Figs. 2 and 3). Thanks to early diagnosis, accurate imaging techniques, and advanced chemotherapy, the joints at each end of the long bone may be preserved to ensure better postoperative functions. The bone defect after tumor resection may be reconstructed by different techniques, as mentioned above. An intercalary prosthesis may be applied to restore limb function immediately after the operation, but the risk of delayed complications such as aseptic loosening, infection, mechanical failure, and fracture of the adjacent bone may be higher than that of biological constructions. Therefore, the use of an intercalary prosthesis is a preferred method for patients with metastatic and multiple myeloma than for those with a primary bone tumor [4].

Biological reconstruction has been widely adopted for the treatment of primary bone sarcomas because it may preserve limb function better and has fewer long-term complications compared with endoprosthesis reconstruction. There are several options for the biological reconstruction of defects in the diaphysial and metadiaphysial regions of long bones, as mentioned above. We choose to use the devitalized tumor-bearing bones because their shapes allows a perfect fit of the tumor resection (Figs. 2d and 3d); the tight contact between the bone ends is an exceptional advantage for bone union compared to allograft (Figs. 2e and 3e). Excellent mechanical properties, low medical cost, and good osteoinductivity also make it a favorable choice, as reported in the literature [6]. One study [12] showed that irradiation, autoclaving, pasteurization, and freezing with liquid nitrogen can all devitalize tumor cells effectively, although the mechanical properties and osteoinduction ability of the devitalized bone may vary. Of these methods, pasteurization has the least effect on the mechanical properties of devitalized bone.

Pasteurization is a proven effective method for devitalizing bones with a tumor, as reported in the literature [6]. On this basis, several researches have been done to investigate the activity of those bone-healing-related proteins in the devitalized bone. The osteoinductivity of human bone morphogenetic protein in the bone may be preserved to some extent during treatment by pasteurization [13, 14], and this may facilitate bone union when this method is chosen for the reconstruction of a bone defect. Clinical and experimental studies have shown that heating is an effective option for devitalizing tumor cells and that the devitalized bone integrates well with the residual bone end. Whether the periosteum is preserved or not is not mentioned in the previous literature. We choose to preserve the periosteum around the tumor-bearing bone when devitalizing bone because the periosteum may serve as a natural structural habitat for mesenchymal stem cells (MSCs) and it provides an essential place for MSCs to harbor and differentiate during the healing process [15]. The preserved factors and the factors secreted around the bone during the inflammatory phase after surgery may recruit MSCs and initiate the bone-healing process.

Salt ions are highly mobile in solution. According to the Hofmeister effect, a high concentration of salt may shield electrostatic interactions in protein, which would further change the spatial conformation of proteins and increase their thermal stability [9, 10]. Hypertonic saline has been widely used as a protein stabilizer and precipitant in biochemistry and molecular biology; so we chose to use hypertonic saline instead of physiological saline as the devitalizing reagent to make a better preservation of protein activity during the pasteurization. Twenty percent saline is the most concentrated saline preparation in our clinical use, and we choose this reagent for devitalization. Sodium chloride is a protein stabilizer rather than a protein denaturant when in a high concentration [9, 16]; thus, the protein activity would not be affected when the high-concentration salt was washed away by normal saline after the devitalization.

No devitalized bone-related recurrence was observed in our study. This confirmed our method to be a very effective and safety method of devitalization. Recurrence occurred in the surrounding soft tissue in three patients and in the residual bone in one patient; the local recurrence rate was 14.8 % in our study. This rate might reflect the narrow operation field and the use of a relatively conservative osteotomy for the sake of better reconstruction with long residual bone ends.

The bone union rate and fracture rate in our study are similar to or better than those reported in the literature (Table 1). This may due to the better protection of the activity of bone-healing-related protein during incubating in 20 % saline and the preservation of periosteum around the devitalized bone. Chen et al. observed a bone nonunion rate of 7 % and a devitalized bone fracture rate of 20 % in their study of 15 patients who received intercalary devitalized bone reconstruction after the recycled bone had received 300 Gy irradiation [17]. Jeon et al. performed intercalary reconstruction with devitalized tumor-bearing bone after pasteurization in 21 patients; the rates of deep infection, bone fracture, and bone nonunion were 14.3, 9.5, and 23.8 %, respectively [6]. But in this article, the authors calculated the bone nonunion rate by patients instead of by bone ends, which make the bone nonunion rate higher than that calculated by bone ends. Pan et al. studied ten patients who received autoclaved autograft implantation, and the rates of nonunion, infection, and devitalized bone fracture were 30, 20, and 20 %, respectively [18].

**Table 1:** Comparison with previous studies

Authors	Number of patients	Devitalized method or graft being used	Nonunion rate (%)	Mean time to union (months)	Deep infection (%)	Fracture (%)	Mean follow-up time (months)
Our study	27	20 % Saline, 65 °C, 30 min	7.4	11	11.1	3.7	62.8
Jeon et al.	21	Pasteurized	23.8	15.5	14.2	9.5	74.3
Chen et al.	15	Irradiation	7	–	0	20	71
Pan et al.	10	Autoclave	30	12	20	20	35
Bus et al.	87	Allograft	40	–	14	29	84
Li et al.	11	Allograft + vascularized fibula graft	9	11.8	0	0	34.1

En dash means data not given

Intercalary allografts are used widely to repair the bone defect after tumor resection. Delayed union and nonunion are not uncommon and may lead to higher rates of mechanical failure and infection [1]. In the paper by Hornicek et al., a nonunion rate of 19.5 % was observed in the 248 patients who received an intercalary allograft implantation. The bone union rates in our study were better than most of those reported for allograft reconstruction in the literature. Ortiz-Cruz et al. reported that 30 % of 103 patients who received an intercalary allograft reconstruction exhibited nonunion after a mean follow-up of 73 months [19]. Bus et al. reported a multicenter study of the use of intercalary allograft reconstructions following resection of primary bone tumors [20]. The nonunion, fracture, and infection rates were 40, 29, and 14 %, respectively. The complication rate was so high that the authors suggested that the use of allografts for reconstruction of defects larger than 15 cm should be reconsidered, especially in older patients.

An allograft combined with an intramedullary vascularized fibula graft was introduced to address an intercalary defect, and this procedure has achieved better results than those with an allograft alone. Li et al. reported a cohort study of 11 patients who received this technique [21]; nonunion occurred in one patient (9 %), and the mean time until weight-bearing was 12.4 months. These results indicate that it is a favorable option to address an intercalary bone defect, but the anastomosis is demanding and the procedure is time-consuming, as noted in the literature.

The induced-membrane technique, also known as the Masquelet technique [7], is suitable for reconstruction of bone defects in the upper limbs. However, it may not suitable for reconstruction of long segmental defects in the lower limbs because the slow bone integration and remodeling might lead to mechanical failure.

Mechanical failure of the internal fixation is not uncommon in patients with intercalary grafting for massive bone defects. Most authors believe that solid fixation would facilitate bone union. Several studies have concluded that stainless steel plates provide more effective support than intramedullary nails or titanium plates [5]. We recommend that the affected limb should be protected with a brace until bone healing is obvious on radiographs.

A limitation of this study is that it was not a randomized controlled study. Patients with different diagnoses, tumor locations, and use of chemotherapy were included in the same group, which made it was impossible to analyze the differences in complications. The preference for using plates versus intramedullary nails and the fact that surgeons performed the procedures without standardized training also made it difficult to determine which fixing option was better. The number of patients was small because we were more aggressive in obtaining a clear margin in the first several years of the study period, and an endoprosthesis replacement might have been performed for a case similar to that shown in Fig. 3.

## Conclusions

Tumors in the middle part of a long bone may be resected without sacrifice joints at both ends. Several options can be used to reconstruct the segmental defect following tumor resection. The devitalized tumor-bearing bone graft may serve as a favorable option, because the bone graft matches the segmental defect well. Our results suggest that devitalization of tumor-bearing bone with 20 % saline at 65 °C for 30 min is an effective method for recycling bone. Favorable union and acceptable complication rates make it a suitable alternative for intercalary reconstruction of segmental bone defects.
